# The Value of Lung Biopsy in Infants up to 3 Months With Diffuse Lung Disease in a Resource‐Limited Setting

**DOI:** 10.1155/carj/1509295

**Published:** 2026-09-01

**Authors:** Pierre Goussard, Pawel Schubert, Lizelle van Wyk, Lynn Booysen, André Gie, Michael Urban, Jacques Janson, Savvas Andronikou, Ernst Eber

**Affiliations:** ^1^ Department of Paediatrics and Child Health, Faculty of Medicine and Health Sciences, Stellenbosch University and Tygerberg Hospital, Cape Town, South Africa, sun.ac.za; ^2^ Department of Pathology, Division of Anatomical Pathology, Tygerberg Hospital, National Health Laboratory Service, Faculty of Medicine and Health Science, Stellenbosch University, Cape Town, South Africa, sun.ac.za; ^3^ Department of Obstetrics and Gynaecology, Faculty of Medicine and Health Sciences, Stellenbosch University and Tygerberg Hospital, Cape Town, South Africa, sun.ac.za; ^4^ Division of Human Genetics, National Health Laboratory Service and School of Pathology, University of the Witwatersrand, Johannesburg, South Africa, wits.ac.za; ^5^ Department of Surgical Sciences, Division of Cardiothoracic Surgery, Faculty of Medicine and Health Sciences, Stellenbosch University and Tygerberg Hospital, Cape Town, South Africa, sun.ac.za; ^6^ Department of Radiology, Children’s Hospital of Philadelphia, Philadelphia, Pennsylvania, USA, chop.edu; ^7^ Department of Radiology, Perelman School of Medicine, University of Pennsylvania, Philadelphia, Pennsylvania, USA, upenn.edu; ^8^ Department of Paediatrics and Adolescent Medicine, Division of Paediatric Pulmonology and Allergology, Medical University of Graz, Graz, Austria, medunigraz.at

**Keywords:** childhood interstitial lung diseases, genetic testing, lung biopsy, pulmonary hypertension, term neonate

## Abstract

**Introduction:**

Neonatal lung biopsy guides management of unusually severe, diffuse lung disease with an uncertain diagnosis. Childhood interstitial lung disease (chILD) constitutes a diverse group of uncommon respiratory diseases which are associated with major morbidity and mortality. The incidence, outcome and mortality of chILD and other severe respiratory diseases in resource‐limited settings (RLS) are unclear.

**Objective:**

This retrospective, descriptive study examined lung biopsy in an RLS on infants up to 3 months of age to diagnose and facilitate management. This study included neonates with severe respiratory distress not responding to surfactant replacement.

**Results:**

Lung biopsy was diagnostic in 94% (29/31) of patients without procedure‐related mortality. The mean gestational age at birth was 35.8 weeks (SD ± 4.6). The mean presentation age was 20 days of life (IQR 1–28). Of 31 participants, 16% (*n* = 5) had histological findings in keeping with pulmonary interstitial glycogenosis (PIG) only. Sixteen percent (*n* = 5) of biopsies showed findings in keeping with adenosine triphosphate (ATP)–binding cassette subfamily A member 3 (ABCA3) deficiency in combination with features of PIG on electron microscopy. Surfactant protein (SFTP)‐B deficiency and undefined surfactant deficiency were equally prevalent (6% (*n* = 2). Pulmonary hypertension (PHT) featured in 61% (*n* = 17) of infants. Genetic testing was performed on 29% (*n* = 9) of infants, with 56% (*n* = 5) showing normal results. The cohort mortality was 42% (*n* = 13).

**Conclusions:**

ChILD should be considered in neonates with severe respiratory distress and PHT not responding to surfactant replacement. Lung biopsy is safe and diagnostic and may prevent long and futile treatment. Histopathological diagnosis frequently assists treatment decisions. Future multicentre studies should include children from an RLS to collect information on diagnoses, genetics and treatment responses.

## 1. Introduction

Lung biopsy in the neonatal period is indicated in unusually severe, diffuse lung disease of uncertain nature, where a definitive diagnosis may guide management decisions, including transition to palliative care in some cases [1, 2].

A lung biopsy often needs to be performed in infants with significant comorbidities [2]. The reported diagnostic yield from lung biopsy is variable, and its utility in bringing about a change in the treatment of patients remains uncertain [3, 4].

Childhood interstitial lung disease (chILD) constitutes a diverse group of uncommon respiratory diseases which are associated with major morbidity and mortality [5]. chILD is increasingly recognised and includes various developmental, genetic, inflammatory, infectious and reactive disorders not identified in adults. The incidence of chILD varies between 0.13 and 10.76/100,000 with a mortality of approximately 15% [6, 7]. The diffuse developmental disorders, growth abnormalities, specific conditions of undefined aetiology (neuroendocrine cell hyperplasia of infancy [NEHI] and pulmonary interstitial glycogenosis [PIG]) and the surfactant protein (SFTP) disorders are seen in the neonate.

The earliest possible presentation of chILD is shortly after birth when the neonate presents with unexplained respiratory distress needing intubation and ventilation. Although not common, chILD patients can be born preterm with acute respiratory distress, which is more severe than would be expected because of prematurity [8].

Adverse sequelae of chILD may extend into adult life if the affected infant survives [9].

The incidence, outcome and mortality of chILD and other severe respiratory diseases in low‐ and middle‐income countries (LMICs) are unclear. There is currently no specific treatment for these conditions, especially those affecting the newborn and management is supportive, often with a poor prognosis. Lung transplant remains the only definitive treatment in many cases [10]. Lung biopsy is often beneficial to avoid prolonged and futile treatment [11]. A specific diagnosis also remains important for counselling as well as future pregnancies, but in LMICs it is often hampered by a lack of diagnostic capabilities [12]. Genetic analysis, which may be diagnostic, can be expensive, time‐consuming and complex.

We describe a cohort of infants, up to 3 months of age, presenting in a resource‐limited settings (RLS), where a lung biopsy was performed, to determine the diagnosis of severe respiratory disease and facilitate further management.

## 2. Patients and Methods

This is a retrospective, descriptive study performed at Tygerberg Hospital, a resource‐limited, public healthcare facility in Cape Town, South Africa. This study was approved by the Health Research Ethics Committee of the Faculty of Medicine and Health Science of Stellenbosch University, HREC approval number N18/01/010. As this is a retrospective review, we received a waiver of individual informed consent for the data, which were routinely collected as part of clinical management by the paediatric service. A paediatric lung biopsy database was used to identify infants who had presented up to 3 months of age with prolonged, severe respiratory diseases and who had lung biopsies performed between 2004 and 2021.

### 2.1. Lung Biopsy Samples

**FIGURE 1:**
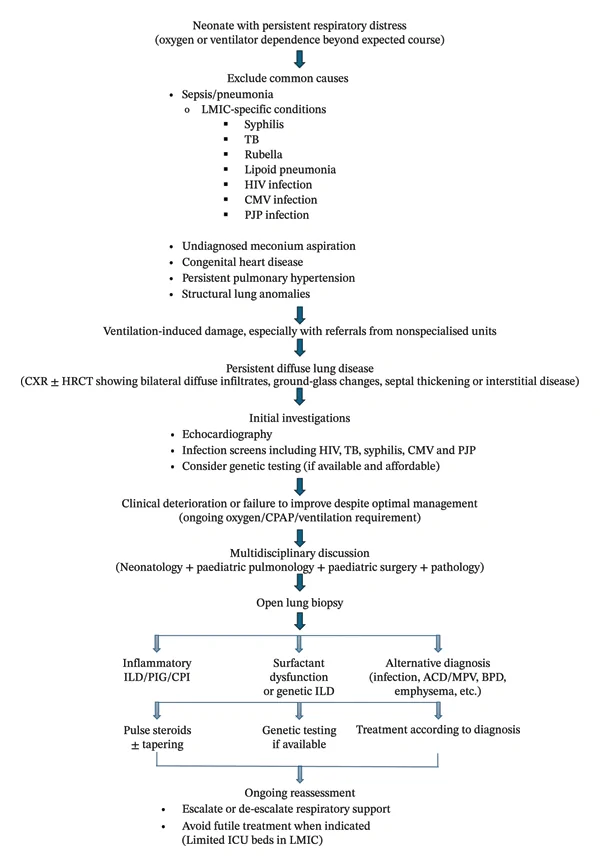
Diagnostic approach in LMICs.

Lung biopsy was considered in infants with persistent or worsening respiratory failure when clinical, radiological or laboratory investigations did not establish a diagnosis. Routine investigations were done before lung biopsy was considered, including relevant investigations for an LMIC setting (Figure 1). A chest CT scan was done routinely before biopsy except in children with high ventilation needs or high‐frequency oscillatory ventilation who were deemed too sick to be transported for a CT scan. Other considerations included a high likelihood that the histopathology findings would influence therapy and prognosis. All patients had an echocardiography performed to exclude cardiac lesions and determine the presence or absence of pulmonary hypertension (PHT). PHT was defined as mean pulmonary artery pressure above 20 mmHg on echocardiography.

Important considerations were the presence of diffuse lung disease on imaging and whether the patient was stable enough for the procedure. The decision was always made by a multidisciplinary team including paediatric pulmonology, neonatology, radiology, pathology and cardiothoracic surgery. The patient was referred for anaesthetic consultation before the final decision to determine risk and if the procedure could be safely done. Lung biopsies were performed during open thoracotomy. The lung biopsies were split after sampling. A section of each biopsy was fixed in 10% buffered formalin, embedded in paraffin wax and stained with haematoxylin and eosin, periodic acid‐Schiff (PAS) and SFTP‐B immunohistochemistry. A section of the biopsied tissue was further fixed with glutaraldehyde for electron microscopy. The ABCA3 diagnosis was based on both histological and EM findings. In this study, ‘chronic pneumonitis of infancy (CPI)’ was used as a descriptive histologic pattern, whereas ‘surfactant deficiency undefined’ was applied when features suggested a surfactant dysfunction disorder but without definitive histologic, ultrastructural, or genetic confirmation of a specific defect. A diagnosis of isolated PIG was reserved for cases in which there was diffuse interstitial glycogen‐laden mesenchymal cell proliferation without accompanying alveolar simplification or other growth abnormalities. In isolated cases of PIG, other developmental and surfactant‐related abnormalities were excluded as humanly possible. All biopsies were initially interpreted at the time of clinical care. For the study, all available histologic material underwent a standardised re‐review by a single experienced paediatric pulmonary pathologist with more than 20 years’ experience in paediatric lung pathology. The stated duration of experience reflects cumulative expertise at the time of re‐review rather than at the time of each original diagnosis. Collected data included demographic data, presenting signs and symptoms, radiological and echocardiographic findings, histology findings and genetic analysis results, as well as clinical outcomes.

### 2.2. Genetic Testing

Diagnostic genetic testing was not routinely available throughout the study period in the South African state/public health sector. In fact, there was very limited genetic testing available during this time period. Most samples were stored and only analysed much later and did not play a role in the acute management of the patients. Management decisions were solely based on the histological findings. Where possible, diagnostic genetic testing was outsourced, usually to a pathology laboratory in the South African private health sector (Ampath Laboratories, Centurion, Pretoria). Next‐generation sequencing (NGS) of panels of 8–14 genes was performed on DNA extracted from blood samples. The panels included genes associated with congenital or childhood‐onset lung disease and in all cases had a minimum of the following genes: ^1^ *ABCA3, NKX2-1, SFTP-A2, SFTP-B, SFTP-C, TERC* and *TERT*. Gene panel tests were validated to have a high detection for variants involving one or a few base pairs but were not optimised for detection of large deletions or duplications of genetic material.

The original diagnosis was based on the histological, including EM, findings. Genetic testing was done at a later stage, as this was not available at the time of the lung biopsy. The original histological diagnosis was amended for this study when there were genetic results available at re‐review.

## 3. Results

**FIGURE 2:**
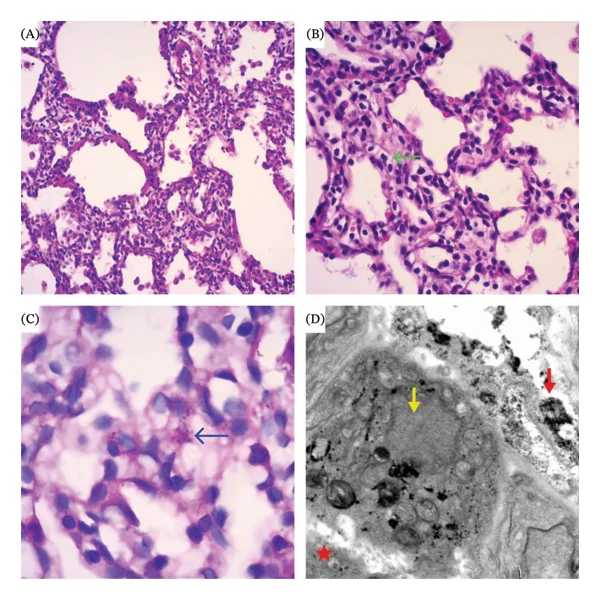
Patient 18: PIG. (A) Intermediate‐power magnification showing the lung where the alveolar septae are expanded (H&E stain, 20*x*). (B) High‐power magnification showing the expanded alveolar septae lined by cuboidal pneumonocytes with a clear cytoplasm and bland ovoid nuclei. No inflammatory cells are seen (H&E stain, 40*x*). (C) PAS stain showing glycogen granules (blue arrow) in the clear interstitial cells (PAS stain, 40*x*). (D) Electron microscopy image with the alveolar lumen present in the lower left corner (marked with an asterisk). The first cells are a Type II pneumocytes (yellow arrow) and the red arrow highlights stromal cells with glycogen in its cytoplasm (electron microscopy tissue was processed with the glycogen protocol which marks glycogen as black).

**FIGURE 3:**
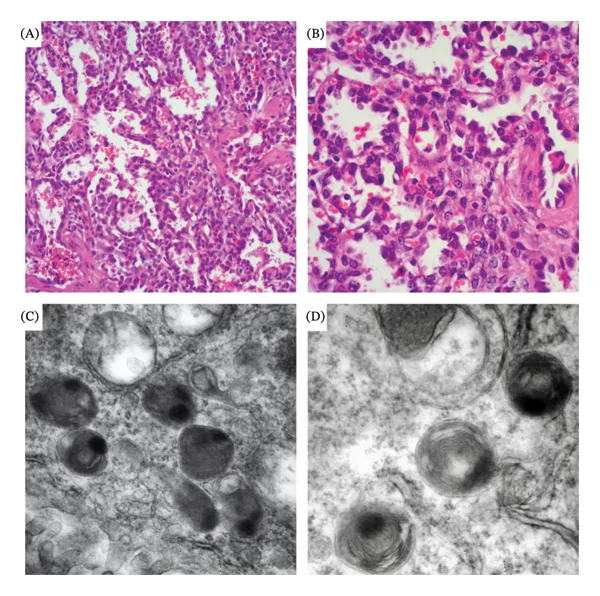
Patient 7: ABAC3. (A) Intermediate‐power magnification image of the lung biopsy showing a lung where the alveolar lumens are clean/empty and the alveolar septae are cellular (H&E stain, 20*x*). (B) High‐power magnification of the lung biopsy with a reaction pattern of chronic pneumonitis of infancy with cellular alveolar septae and reactive‐type pneumocytes lining the alveolar walls. The surfactant immunostain for surfactant B was positive (not shown). (C and D) show electron microscopy images of small, tightly wound surfactant lamellar bodies giving a fingerprint‐like appearance with an electron‐dense inclusion in the lamellar body.

**FIGURE 4:**
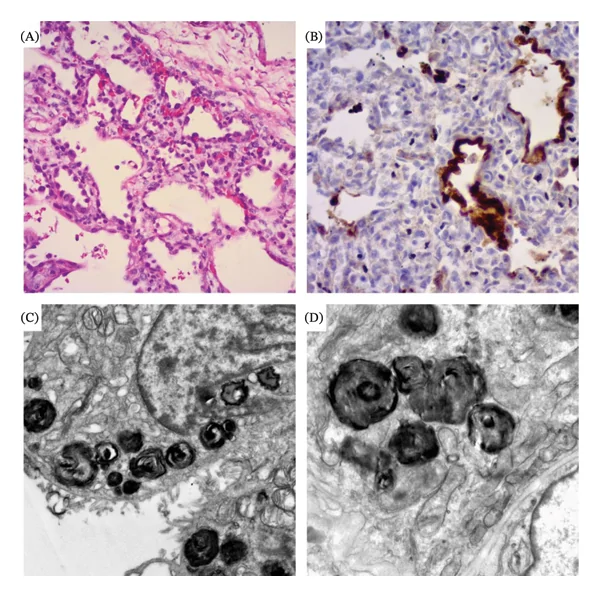
Patient 5: ABCA3 NOVEL. (A) High‐power image from the lung biopsy, showing alveolar ducts lined by Types I and II pneumocytes with expanded alveolar septae that appear somewhat cellular. (B) Immunocytochemical stain for surfactant B showing some alveolar pneumocytes staining positive, while some are negative (left side of image). Also note the hypercellular alveolar septae again giving a chronic pneumonitis of infancy reaction pattern (SPB immunostain, 40*x*). (C and D) Electron microscope image of the surfactant lamellar bodies showing an abnormal morphology with electron‐dense inclusions, with some demonstrating a tightly wound lamellar appearance (in D), while in C the lamellae have spaces between them (which would be more of a normal feature). This case had genetic results of a novel ABCA3 mutation.

**FIGURE 5:**
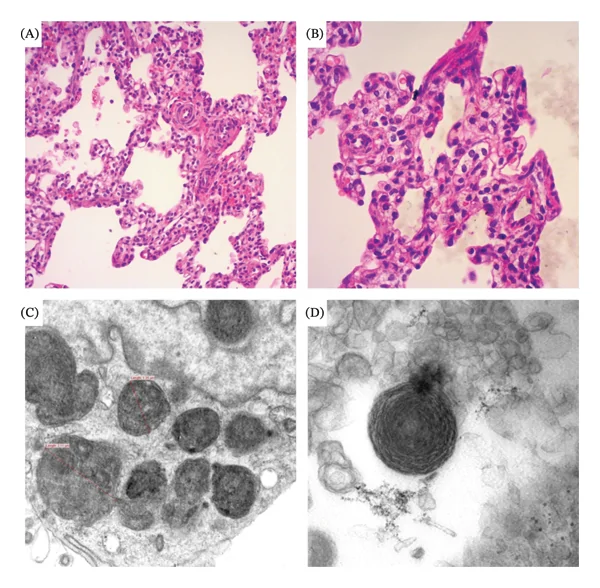
Patient 16: PIG and ABCA3—no mutation. (A) Demonstrates lung with widened interstitial septa but empty alveolar spaces (H&E stain 20*x*). (B) Shows a high magnification of the interstitium with clear cells that were shown to contain glycogen on the PAS and PASD stains (H&E stain, 4ox). (C and D) Show electron microscopic images of the surfactant bodies. The sizes varied from 600 to 1700 nm and appeared to have tightly wound lamellae, with some containing an electron‐dense area. These lamellar bodies do not have the classic appearance of normal lamellar bodies but rather show a kingship to those found in ABCA3 deficiency (compare to Figures 2 and 3). The sizes of the lamellar bodies varied from small to normal ranges. Genetics found no abnormalities.

**FIGURE 6:**
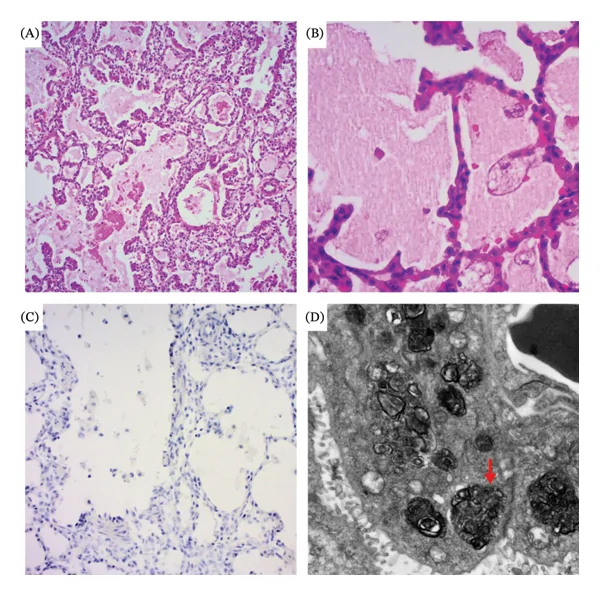
Patient 6: SPB deficiency. (A) Intermediate‐power magnification from the lung biopsy showing alveolar structures that are filled with an eosinophilic material. The alveolar septae show a normal cellularity for age (H&E stain, 10*x*). (B) High‐power magnification showing alveoli that are lined by Type I pneumonocytes and filled with an acellular, granular material compatible with features of alveolar proteinosis (H&E stain, 40*x*). (C) Surfactant B immunohistochemical stain showing no staining in the pneumocytes of alveolar macrophages (20*x*). (D) Electron microscope image showing multivesicular lamellar bodies with very distorted lamellar architecture and vacuolisation and irregular in size.

**FIGURE 7:**
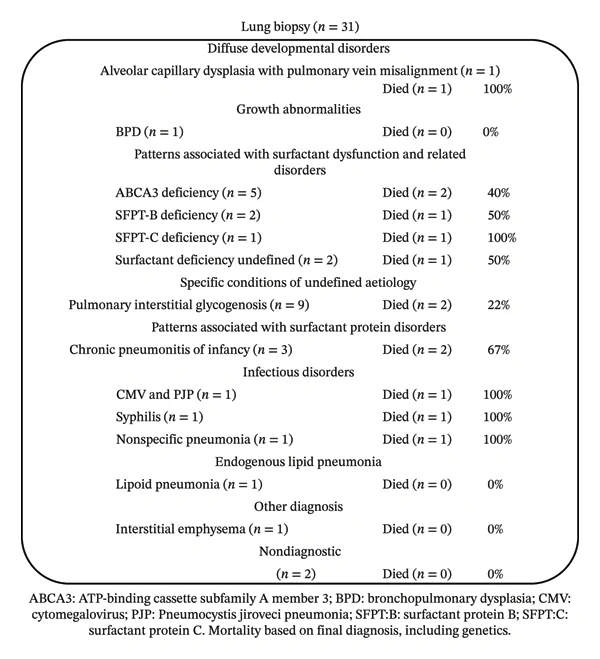
Lung disease and mortality according to biopsy and genetic results.

Lung biopsy was performed at a median postnatal age of 46 days and was diagnostic in 94% (*n* = 29). Some lung biopsy findings overlapped (Table 1). PIG was seen in 39% (*n* = 12) of biopsies (Figure 2A–D). A combination of PIG and adenosine triphosphate (ATP)–binding cassette subfamily A member 3 (ABCA3) deficiency was present in 16% (*n* = 5), and a combination of PIG with bronchopulmonary dysplasia (BPD) in 6% (*n* = 2). Isolated PIG was histologically present in 16% (*n* = 5). Pneumonic changes were seen in 10% (*n* = 3) and were due to: (1) a combination of cytomegalovirus (CMV) and *Pneumocystis jiroveci* (PJP) infections in an HIV‐positive infant; (2) features in keeping with syphilitic pneumonia in an infant with congenital syphilis; and (3) bacterial pneumonia (blood culture positive for Acinetobacter). Two cases each were found of ABCA3 deficiency (Figure 3A–D, Figure 4A–D and Figure 5A–D), SFTP B deficiency (Figure 6A–D) and undefined surfactant deficiency, as was diagnosed with electron microscopy. CPI was diagnosed in 10% (*n* = 3). Other biopsy results showed single cases of alveolar capillary dysplasia (ACD) with misalignment of pulmonary veins (ACD/MPV), alveolar proteinosis, BPD and interstitial emphysema. Inconclusive lung biopsy findings were present in 6% (*n* = 2) (Figure 7).

**TABLE 1:** Diagnoses, treatment and outcomes.

Final diagnosis	Histological findings	Genetic testing results	Case no.	Sex	Birth gestation (weeks)	Birth weight (grams)	RVDE	Age at presentation (days)	Length of initial hospital stay (days)	Length of ICU stay (days)	Year of biopsy	Age at biopsy (days)	CT scan findings	Pulmonary arterial hypertension (PAH)	Maximum respiratory support required	Steroid pulsed therapy	Final outcome	Value of biopsy
Surfactant defects	PIG and ABCA3 deficiency	ABCA3 deficiency	1	F	40	3700	N	1	349	349	2015	15	Ground glass/septal thickening	Yes	Ventilation	Yes	Died	No active treatment
	CPI	SFTP‐C deficiency	4	M	37	2000	N	22	73	56	2013	90	Ground glass/septal thickening	Yes	Ventilation	Yes	Died	Continuation of treatment and reintubation
	ABCA3 deficiency	ABCA3 deficiency	5	M	37	2190	N	1	52	35	2013	43	Not done	No	Ventilation	Yes	Died	No active treatment
	SFTP‐B deficiency	SFTP‐B deficiency	6	M	35	1920	Y	1	111	15	2014	46	Diffuse reticular/cystic	Yes	CPAP	Yes	Died	CPAP but not for ventilation
	ABCA3 deficiency	—	7	F	40	2720	N	6	84	13	2015	56	Ground glass/septal thickening	No	CPAP	Yes	Survived	CPAP but not for ventilation
	Surfactant deficiency undefined	—	8	M	40	3160	N	42	43	4	2016	89	Ground glass/septal thickening/opacification	No	CPAP	No	Survived	CPAP but considered for ventilation
	SFTP‐B deficiency	—	11	M	40	3340	N	1	28	14	2017	17	Opacification	Yes	Ventilation	Yes	Survived	Weaned from ventilation but not considered for reintubation
	PIG and ABCA3 deficiency	—	12	M	36	2000	Y	19	22	8	2018	34	Interstitial	No	Ventilation	Yes	Survived	Weaned from ventilation but not considered for reintubation
	PIG and ABCA3 deficiency	—	13	M	31	1780	Y	45	16	3	2013	66	Ground glass/opacification	No	CPAP	Yes	Survived	Weaned from CPAP but not considered for reintubation
	Surfactant deficiency undefined	—	30	F	31	1410	Y	1	4	2	2020	5	Not done	Yes	Ventilation	No	Died	Continuation of treatment/died on ventilation

PIG	PIG	Not pathogenic	9	M	40	3800	N	26	31	31	2017	21	Not done	Yes	Ventilation	Yes	Died	Continuation of treatment/died on ventilation
	PIG and ABCA3 deficiency	Not pathogenic	10	M	29	1520	N	1	119	63	2017	59	Ground glass	Yes	Ventilation	Yes	Survived	CPAP but not considered for ventilation
	PIG and alveolar growth abnormalities	—	14	F	40	3060	N	19	19	1	2015	30	Not done	No	Nasal prong oxygen	Yes	Survived	Continuation of treatment and considered for reintubation
	PIG and alveolar growth abnormalities	—	15	F	40	1900	N	1	90	62	2014	77	Interstitial/septal thickening/opacification	Yes	Ventilation	Yes	Died	Continuation of treatment/died on ventilation
	PIG	—	16	M	37	2890	N	1	367	336	2015	14	Ground glass	Yes	Ventilation	Yes	Survived	Continuation of treatment/died on ventilation
	PIG	—	17	M	30	1160	N	93	35	9	2016	120	Ground glass/opacification	No	CPAP	Yes	Survived	CPAP but considered for ventilation
	PIG	—	18	M	40	4030	N	1	42	17	2013	58	Opacification	No	Ventilation	Yes	Survived	Continuation of treatment and considered for reintubation
	PIG	Not pathogenic	29	M	26	790	N	1	365	358	2015	82	Not done	Yes	Ventilation	Yes	Survived	Continuation of treatment and considered for reintubation
	PIG and ABCA3 deficiency	Not pathogenic	31	M	40	3810	N	1	3M	9	2018	12	Not done	Yes	Ventilation	Yes	Survived	Continuation of treatment/considered for reintubation

Pneumonia	Lipoid pneumonia	—	19	F	40	3000	N	76	61	0	2012	90	Opacification	No	Nasal Prong Oxygen	No	Survived	education and remove source
	Pneumonia/abscess	—	24	M	40	4460	N	1	52	52	2014	33	Not done	Yes	Ventilation	No	Died	Continuation of treatment/died on ventilation
	Pneumonia	—	25	M	33	2700	Y	87	31	18	2009	92	Not done	Not done	Ventilation	No	Died	Treatment for PJP and CMV
	Syphilis	—	27	M	38	3600	N	44	1	1	2015	44	Not done	Not done	Ventilation	No	Died	Treatment of syphilis

CPI	CPI	Not pathogenic	3	F	29	1100	N	91	365	5	2010	84	Ground glass/septal thickening	Yes	Ventilation	Yes	Died	Continuation of treatment and reintubation
	CPI	—	21	F	36	2460	N	1	61	4	2014	72	Interstitial	Yes	CPAP	Yes	Survived	CPAP but considered for ventilation
	CPI	—	22	M	35	2300	N	1	18	18	2018	18	Not done	Yes	Ventilation	No	Died	Continuation of treatment/died on ventilation

Other	ACD/MPV	—	2	M	40	2680	N	9	2	2	2017	10	Not done	Yes	Ventilation	No	Died	No active treatment
	BPD	—	23	M	28	1020	N	1	90	30	2014	31	Cystic	Not done	Ventilation	No	Survived	Continuation of treatment and considered for reintubation
	Interstitial emphysema	—	28	F	29	890	N	1	147	1	2018	122	Cystic	No	CPAP	No	Survived	CPAP but considered for ventilation

Inconclusive	Inconclusive	—	20	M	34	2770	N	28	35	6		49	Opacification/interstitial	No	Ventilation	Yes	Survived	Continuation of treatment and considered for reintubation
	Inconclusive	—	26	M	41	2900	N	1	50	4	2014	23	Not done	Yes	Ventilation	No	Survived	Continuation of treatment and considered for reintubation

*Note:* ABCA3: ATP‐binding cassette subfamily A member 3; ACD/MPV: alveolar capillary dysplasia with misalignment of pulmonary veins; BPD: bronchopulmonary dysplasia; CMV: cytomegalovirus; RVDE: retroviral disease exposure; SFTP‐B: surfactant protein B; SFTP‐C: surfactant protein C.

Abbreviations: CPAP, continuous positive airway pressure; CPI, chronic pneumonitis of infancy; PIG, pulmonary interstitial glycogenosis; PJP, *Pneumocystis jiroveci* pneumonia.

Genetic testing was not freely available during the study period, with very limited testing during the first 10 years of the study period. Genetic testing was only performed in 29% (*n* = 9) of infants, with 56% (*n* = 5) having normal results. In four patients, the genetic test result was considered to fully explain the patient phenotype (Table 2): ABCA3 deficiency was identified in two cases, and SFTP‐B and SFTP‐C deficiency in one case each. The two patients with ABCA3 deficiency were both compound heterozygous, with one of the variants in each patient being the NM_001089.3(*ABCA3*):c.316C>T (p.Arg106Ter) variant. The patients were not known to be related to each other, raising the possibility that this variant is present at increased frequency in the local community.

**TABLE 2:** Results of NGS gene panel tests in nine patients.

Case	Lung biopsy/indication	Variants detected on NGS gene panel test	Zygosity of variant	Interpretation of variant	Genotype–phenotype correlation
1	PIG and ABCA3 deficiency	NM_001089.3(*ABCA3*):c.128G>A (p.Arg43His)	Heterozygous	Multiple assertions on Clinvar [40] as pathogenic	ABCA3 deficiency is an autosomal recessive disorder. The genetic test result is consistent with compound heterozygosity and is considered to fully explain the phenotype
		NM_001089.3(*ABCA3*):c.316C>T (p.Arg106Ter)	Heterozygous	Nonsense mutation is expected to exert a loss‐of‐function effect. Single assertion on Clinvar as pathogenic	

3	CPI	No clinically relevant variants detected	—	—	No genetic cause detected

4	CPI	NM_001317778.2(*SFTP-C*):c.435G>A (p.Gln145=)	Heterozygous	Two assertions on Clinvar, one as pathogenic and one as likely pathogenic	SFTP‐C deficiency is an autosomal dominant disorder, and the presence of a single pathogenic variant is considered to fully explain the phenotype

5	ABCA3 deficiency	NM_001089.3(*ABCA3*):c.316C>T (p.Arg106Ter)	Heterozygous	Nonsense mutation is expected to exert a loss‐of‐function effect. Single assertion on Clinvar as pathogenic	ABCA3 deficiency is an autosomal recessive disorder. The genetic test result is consistent with compound heterozygosity and is considered to fully explain the phenotype
		NM_001089.3(*ABCA3*):c.2210del (p.Lys738Argfs ∗ 23)	Heterozygous	Frameshift variant are expected to exert a loss‐of‐function effect. Not previously reported	

6	SFTP‐B deficiency	NM_000542.5(*SFTP-B*):c.883C>T (p.Arg295Ter)	Homozygous	Nonsense mutation is expected to exert a loss‐of‐function effect. Single assertion on Clinvar as pathogenic.	Surfactant protein B deficiency is an autosomal recessive disorder. Homozygosity for a loss of function variant is considered to fully explain the phenotype

9	PIG	No clinically relevant variants detected	—	—	No genetic cause detected

10	PIG and ABCA3 deficiency	No clinically relevant variants detected	—	—	No genetic cause detected. However, given the evidence for ABCA3 deficiency on lung biopsy, the negative genetic test does not exclude primary ABCA3 deficiency

29	Surfactant deficiency undefined	No clinically relevant variants detected	—	—	No genetic cause detected

31	PIG and ABCA3 deficiency	No clinically relevant variants detected	—	—	No genetic cause detected. However, given the evidence for ABCA3 deficiency on lung biopsy, the negative genetic test does not exclude primary ABCA3 deficiency

*Note:* ABCA3: ATP‐binding cassette subfamily A member 3; SFTP‐B: surfactant protein B; SFTP‐C: surfactant protein C.

Abbreviations: CPI, chronic pneumonitis of infancy; PIG, pulmonary interstitial glycogenosis.

The final diagnosis was based on combined lung biopsy and genetic test results (Table 2). Biopsy in three cases showed PIG and ABCA3 deficiency characteristics on electron microscopy. In two of these three cases, genetic testing confirmed the pathological diagnosis, while in the third case, no genetic abnormalities were found, and the final diagnosis was PIG. However, we recognise that lack of detection of causative genetic variants does not completely exclude the possibility of ABCA3 deficiency.

### 3.1. Adverse Events Related to Lung Biopsy

There were no significant complications or mortality directly related to lung biopsy. Two infants developed a pneumothorax, of which one required prolonged intercostal drainage prior to resolution.

### 3.2. Clinical Outcome and Postbiopsy Management

The median duration of ICU stay was 31 days and of hospital stay 31 days (Table 3). The mortality of the cohort was 42% (*n* = 13). Congenital SFTP defects represented 38% (*n* = 5) of deaths (Table 1). The median age at death was 52 days. The incidence of mortality within the first 3 months of age was 46% (*n* = 6): ABCA3 deficiency (*n* = 1), pneumonia (*n* = 2), PIG (*n* = 1), ACD/MPV (*n* = 1) and undefined surfactant deficiency (*n* = 1). Mortality after 3 months of age occurred in 64% (*n* = 7): SFTP‐C deficiency (*n* = 1), SFTP‐B deficiency (*n* = 1), ABCA3 deficiency (*n* = 1), PIG (*n* = 1), CPI (*n* = 2) and pneumonia (*n* = 1). One infant with ABCA3 deficiency died at 2 years of age after being managed at home with long‐term ventilation via a tracheostomy. Histopathology resulted in a diagnosis not compatible with life, in the absence of lung transplantation, in 16% (*n* = 5) cases.

**TABLE 3:** Demographics, clinical details, ICU stay and outcome.

Clinical variable	*n* = 31
Male, *n* (%)	22 (71)
Gestational age, weeks, mean ± SD	35.8 ± 4.6
Prematurity (< 37 weeks), *n* (%)	14 (45)
Birth weight, g, mean ± SD	2485 ± 998
Very low birth weight (< 1500 g), *n* (%)	6 (19)
HIV‐exposed, *n* (%)	5 (16)
HIV‐positive, *n* (%)	1/16 (6)
Congenital syphilis, *n* (%)	1 (3)
Day of life at presentation, days, median (IQR)	20 (1–28)
ICU stay duration, days, median (IQR)	31 (4–35)
Length of hospitalisation, days, median (IQR)	31 (28–90)
Mortality, *n* (%)	13 (42)
Age at death, days, median (IQR)	52 (44–136)

*Note:* RVD: retroviral disease; IQR: interquartile range.

Abbreviations: ICU, intensive care unit; SD, standard deviation.

Lung biopsy impacted clinical management in multiple domains, including modification of treatment and decisions regarding current and future ventilatory support. Antibiotic therapy was altered in 6% of cases (*n* = 2), and in one patient, exogenous lipoid pneumonia was identified as the underlying aetiology. Treatment was withdrawn in 10% of cases (*n* = 3). In 19% (*n* = 6), patients were not considered suitable candidates for future ventilation. Continuous positive airway pressure (CPAP) was initiated in 16% of cases (*n* = 5); among these, mechanical ventilation would have been pursued if required in 20% (*n* = 1), whereas in the remaining 80% (*n* = 4), escalation to ventilation was not deemed appropriate (Table 3).

Oxygen dependency was seen in 28% (*n* = 5) of survivors at 3 months of age.

PHT was present in 61% (*n* = 17) of infants. All patients with PHT were started on inhaled nitric oxide when on ventilators, which were weaned and changed to oral sildenafil.

Corticosteroids were used in 74% (*n* = 23) of infants postlung biopsy. Corticosteroids were used after lung biopsy if contraindications such as congenital or acquired infections were excluded. The criteria to decide to use corticosteroids were aimed to improve oxygenation, decrease ventilator dependence, decrease oxygen dependence and possibly stop ongoing inflammation (Table 1). As per departmental protocol, methylprednisolone pulses (10 mg/kg) were administered for three consecutive days at monthly intervals. Corticosteroid use included methylprednisolone pulse therapy (*n* = 20); infants with BPD and inconclusive biopsy results received short‐course dexamethasone (*n* = 2); and the infant with PJP received oral prednisolone (*n* = 1). The number of methylprednisolone pulse therapy doses was determined according to the response and survival of the infants. The following number of doses were given: three doses (*n* = 13), two doses (*n* = 4) and one dose (*n* = 1). Azithromycin three times per week was used in four infants, and hydroxychloroquine, at 10 mg/kg daily, in two infants.

## 4. Discussion

This is the first data from the Western Cape, South Africa, to show the various causes of chILD in infants ≤ 3 months of age, as diagnosed by lung biopsy, demonstrating a high diagnostic yield. The most common histologic chILD diagnoses were PIG and ABCA3 deficiency. The spectrum of diseases in this study was diverse but similar to that reported in previous studies using the chILD classification [13, 14]. Mortality of the cohort was high, with a high prevalence of BPD (unrelated to prematurity) and PHT in the survivors.

In the current study, open lung biopsy was performed in a very selective group of infants not responding to ventilation or other respiratory support measures in a resource‐restricted hospital in South Africa. All infants had severe respiratory distress requiring either mechanical ventilation or other respiratory support prior to lung biopsy. Despite this, few adverse effects and no mortality were directly associated with the biopsy procedure.

The clinical definition of chILD requires three out of four of the following criteria to be met in the absence of known disorders: (1) respiratory symptoms (e.g., cough, rapid and/or difficult breathing, or exercise intolerance); (2) respiratory signs (e.g., resting tachypnoea, adventitious sounds, retractions, digital clubbing, failure to thrive, or respiratory failure); (3) hypoxaemia; and (4) diffuse abnormalities on CXR or CT scan [8, 15]. This study’s cohort presented mostly with hypoxaemia, tachypnoea and subcostal retractions. This finding is in keeping with other studies where the most common signs and symptoms were tachypnoea and hypoxia [15]. These persistent symptoms prompted CT scan and lung biopsy, as recommended by international guidelines [8, 15].

In this study, just under half of the population was preterm. This may lead to chILD not being considered due to the assumption of prematurity‐related pulmonary diseases. However, the most common final diagnoses were PIG and ABCA3 deficiency abnormalities, both of which are mostly detected in term infants [16, 17]. chILD should be considered in preterm infants, especially when respiratory disease is progressive with rapid deterioration [18].

The most common chest x‐ray findings were diffuse alveolar changes (32%) and interstitial changes (35%) in this cohort (Table 4).

**TABLE 4:** Radiological findings of patients: chest x‐rays and chest CT scans.

Investigation	Finding	Data
CXR (*N* = 31) (%)	Small lung fields	3 (10)
	Large lung fields	3 (10)
	Diffuse alveolar pattern	10 (32)
	Ground glass opacities	3 (10)
	Interstitial pattern	11 (35)
	Cysts	3 (10)
	Opacity/consolidation	6 (19)

CT scan (*N* = 19) (%)	Interstitial pattern	4 (21)
	Ground glass opacities	9 (47)
	Opacity	8 (42)
	Septal thickening	6 (32)
	Cysts	3 (16)

Abbreviations: CT, computed tomography; CXR, chest x‐ray.

Despite CT being considered the cornerstone of chILD diagnosis [18], only two‐thirds of the current cohort underwent CT scans. This was due to the instability of many infants, precluding their transport to radiology for CT scans. Ground glass opacities were the most common abnormality found on chest CT scans in 47% of cases, but this was only seen in 10% of cases on the plain chest x‐rays (Table 4).

Most infants underwent echocardiography to determine the presence of PHT, as recommended by international protocols [7, 14]. A substantial portion of surviving infants was diagnosed with PHT (41%) due to their disease. PHT is estimated to occur in 1%–64% of chILD patients [19].

Lung biopsies were highly diagnostic. The surfactant dysfunction group had a high mortality, consistent with international data [20].

PIG was identified in just over one‐third of biopsies in this study, with more than half of biopsies also showing features of other abnormalities (ABCA3 deficiency, BPD). PIG has become increasingly recognised in biopsies since it was initially described in 2002 [21, 22].

Infants with lung biopsies showing histopathological features of diffuse PIG have been successfully treated with corticosteroids [21, 23]. Patchy PIG findings on lung histology have been reported in up to 40% of cases classified as having CLD not related to prematurity [24, 25]. In cases with patchy PIG, treatment with corticosteroids is not recommended due to the negative association with postnatal alveolarisation [25]. Mortality in infants with diffuse PIG may be more related to underlying comorbidities, including cardiac disease or prematurity, rather than lung disease per se [21, 26, 27]. In the current study we were unable to differentiate between diffuse or patchy PIG. In the current study, all cases of isolated and combined PIG were treated with steroids, and mortality was low (25%).

The lung biopsy results significantly influenced clinical management, guiding decisions related to treatment, withdrawal of care and both current and future ventilation plans. These decisions were based solely on histological findings, as genetic test results were significantly delayed.

In LMICs, where neonatal ventilation resources are limited, it is crucial to avoid using these scarce beds for futile cases or for prolonged ventilation with poor long‐term outcomes. In this study, ventilation was actively withdrawn in 10% of cases, and in 19% of survivors no further ventilation support was offered.

Unexpected biopsy diagnoses included pneumonia due to PJP, CMV, syphilis and lipoid pneumonia. These are conditions requiring specific treatments that would likely not have been initiated without biopsy confirmation.

Genetic testing is recommended in paediatric patients of all ages with chILD [18]. However, the yield is often relatively low (approximately 22%). This refers to all age groups and is not specific to infants less than 3 months, as in this study [28]. In LMICs, genetic testing is not always available, as it is expensive or may have relatively limited coverage of the relevant genes and may have long test turnaround times, causing diagnostic delays [15]. Although genetic testing was only performed in a third of study patients, the yield was higher (44%) compared to international data [18]. This may be explained by the fact that genetic testing was performed only on carefully selected cases based on lung biopsy results—because of cost and because the panel covered mainly genes related to surfactant metabolism. It is now recognised that several hundred genes can be associated with chronic respiratory disorders in infancy—testing a broader set of genes facilitates the less targeted approach that is currently recommended.

Studies have shown infants had prolonged stays in both NICU and hospital, which affects hospital costs. A stepwise approach to chILD may be beneficial to shorten hospitalisation and preserve hospital resources, especially in resource‐constrained environments.

In this selected group of below 3 months, the mortality was higher than international figures, but the reported chILD‐related mortality is estimated around 15% in all ages of children [2, 6, 29, 30]. This may be due to the relatively high incidence of prematurity as well as the higher prevalence of surfactant deficiencies.

Similar to the high incidence of surfactant disorders in the current cohort, the chILD classification shows predominantly genetic and lung developmental disorders as the causes of ILD among neonates and infants [31].

PIG, which may present with hypoxaemic respiratory insufficiency and PHT in infants, can only be diagnosed with lung biopsy [21]. ILD, due to genetic disorders with surfactant dysfunction, should be suspected in term or near‐term neonates with severe, persistent respiratory failure not improving after 7–10 days and after exclusion of infection and congenital cardiac anomalies [13, 14, 32].

ACD can present with neonatal respiratory failure, severe PHT and hypoxaemia. Chest radiograph findings of ACD are nonspecific, and extra‐pulmonary disorders are seen in more than 50% of infants [33–35]. Lung biopsy remains the gold standard for diagnosing these.

There are limited reports on the use of lung biopsy in the diagnosis of persistent lung disease in neonates and young infants and no reports from LMICs [36]. When either preterm or term infants have an unusually severe diffuse lung disease of uncertain nature, a lung biopsy in the neonatal period is clinically indicated to help guide management decisions, including the possibility of transition to palliative care [37].

In this study corticosteroids were used after lung biopsy if contraindications such as congenital or acquired infections were excluded. Criteria to decide to use corticosteroids were the aims to improve oxygenation, decrease ventilator dependence, decrease oxygen dependence and possibly stop ongoing inflammation.

Although morbidity and mortality from paediatric lung biopsies are reported to be high, in this study there was no procedural mortality [4, 38, 39]. The results would support the relative safety of lung biopsy as a diagnostic investigation even in a critically ill infant.

This is the first study from an LMIC describing the diagnostic yield and findings of histology for chILD. While PIG and ABCA3 may be most commonly detected in term infants, this presumably reflects a decreased level of lung biopsy in patients who have known BPD. This apparent predominance in term infants likely reflects biopsy selection bias rather than true disease prevalence.

The study was limited by both its retrospective nature, the limited number of chest CT scans and the fact that genetic testing was not available for all cases. Cases of ABCA3 were diagnosed on histology with negative genetic testing, but these were re‐evaluated and confirmed to have histology compatible with ABCA3. The limitation remains that ABCA3 deficiency cannot be excluded with complete certainty in genetically negative cases, even when histopathological findings are suggestive. Current genetic testing may not detect all pathogenic variants, and the spectrum of abnormalities in the African setting is not entirely known.

The histological features associated with ABCA3 deficiency are not entirely specific and may overlap with other disorders of surfactant metabolism. The spectrum of electron microscopic variations of the lamellar bodies has not been fully evaluated. Additionally, it is not known what effect different allele mutations in the ABCA3 genes could have on the lamellar body appearance. Hence, in the absence of balletic pathogenic or likely pathogenic ABAC3 variants, the diagnosis of ABCA3 deficiency cannot be definitively established or excluded. Further, it was not possible to interpret treatment responses for individual chILD diagnoses due to the small number of patients.

## 5. Conclusions

Neonatal ILD should be included in the differential diagnosis of all neonates, including in LMICs, especially those born at term, with severe respiratory distress syndrome and PHT not responding to surfactant replacement. ABCA3 deficiency was the most commonly identified surfactant defect in this study, and PIG was seen in combination with other conditions. Lung biopsy was shown to have few complications, and when diagnostic in suspected chILD frequently assisted with treatment decisions, including the avoidance of prolonged and futile treatment. The access to genetic testing has improved, but results are not immediately available, making lung biopsy essential in many instances. Due to the small number of cases seen in individual centres, future multicentre studies should include children from resource‐limited countries.

## Author Contributions

Pierre Goussard and Lizelle van Wyk conceived and designed the research. Pierre Goussard, Lizelle van Wyk and Lynn Booysen interpreted the data and drafted the manuscript. Pierre Goussard, Lizelle van Wyk, Pawel Schubert, Michael Urban, Jacques Janson, Savvas Andronikou, André Gie and Ernst Eber contributed to critical review and editing the manuscript.

## Funding

This study was not funded.

## Disclosure

The research for this study was done in partial fulfilment of the requirements for the MMed (Paed) degree at Stellenbosch University under Dr Lynn Booysen [41]. All the authors approved the final version of the manuscript. All authors agreed to be accountable for all aspects of the work.

## Conflicts of Interest

The authors declare no conflicts of interest.

## Data Availability

The data that support the findings of this study are available on request from the corresponding author. The data are not publicly available due to privacy or ethical restrictions.

## References

[1] Griese M. , Irnstetter A. , Hengst M. et al., Categorizing Diffuse Parenchymal Lung Disease in Children, Orphanet Journal of Rare Diseases. (September 2015) 10, no. 1, 10.1186/s13023-015-0339-1. PMC4582630 26408013

[2] Griese M. , Haug M. , Brasch F. et al., Incidence and Classification of Pediatric Diffuse Parenchymal Lung Diseases in Germany, Orphanet Journal of Rare Diseases. (December 2009) 4, no. 1, 10.1186/1750-1172-4-26. PMC2800105 20003372

[3] Lamoshi A. Y. and Nakayama D. K. , Usefulness of Lung Biopsy in Pediatric Pulmonary Conditions, The American Surgeon. (January 2015) 81, no. 1, 31–33, 10.1177/000313481508100121. 25569057

[4] Naiditch J. A. , Barsness K. A. , and Rothstein D. H. , The Utility of Surgical Lung Biopsy in Immunocompromised Children, Journal of Pediatrics. (2013) 162, no. 1, 133–136, 10.1016/j.jpeds.2012.06.019. 22817907

[5] Semple T. R. , Ashworth M. T. , and Owens C. M. , Interstitial Lung Disease in Children Made Easier Well, Almost, Radiographics. (October 2017) 37, no. 6, 1679–1703, 10.1148/rg.2017170006. 29019755

[6] Griese M. , Schwerk N. , Carlens J. et al., Minimal Important Difference in Childhood Interstitial Lung Diseases, Thorax. (May 2023) 78, no. 5, 476–483, 10.1136/thorax-2022-219206. 36572533 PMC10176404

[7] Klay D. , Platenburg M. G. J. P. , van Rijswijk R. H. N. A. J. , Grutters J. C. , and van Moorsel C. H. M. , ABCA3 Mutations in Adult Pulmonary Fibrosis Patients: A Case Series and Review of Literature, Current Opinion in Pulmonary Medicine. (May 2020) 26, no. 3, 293–301, 10.1097/MCP.0000000000000680. 32238781

[8] Bush A. , Cunningham S. , de Blic J. et al., European Protocols for the Diagnosis and Initial Treatment of Interstitial Lung Disease in Children, Thorax. (November 2015) 70, no. 11, 1078–1084, 10.1136/thoraxjnl-2015-207349. 26135832

[9] Nathan N. , Corvol H. , Amselem S. , and Clement A. , Biomarkers in Interstitial Lung Diseases, Paediatric Respiratory Reviews. (September 2015) 16, no. 4, 219–224, 10.1016/j.prrv.2015.05.002. 26027849

[10] Eldridge W. B. , Zhang Q. , Faro A. et al., Outcomes of Lung Transplantation for Infants and Children With Genetic Disorders of Surfactant Metabolism, Journal of Pediatrics. (May 2017) 184, 157–164.e2, 10.1016/j.jpeds.2017.01.017. 28215425 PMC5443678

[11] Bush A. , Gilbert C. , Gregory J. , Nicholson A. G. , Semple T. , and Pabary R. , Interstitial Lung Disease in Infancy, Early Human Development. (November 2020) 150, 10.1016/j.earlhumdev.2020.105186. 32958330

[12] Restrepo S. M. , Ojeda P. , Garcia J. , Caceres J. , Rodriguez-Martinez C. E. , and Nino G. , Targeted Immunostaining for the Detection of Children’s Interstitial Lung Diseases (ChILDs) in Low-Middle Income Countries, American Journal of Respiratory and Critical Care Medicine. (2014) 189.

[13] Whitsett J. A. , Wert S. E. , and Weaver T. E. , Alveolar Surfactant Homeostasis and the Pathogenesis of Pulmonary Disease, Annual Review of Medicine. (2010) 61, no. 1, 105–119, 10.1146/annurev.med.60.041807.123500. PMC4127631 19824815

[14] Gupta A. and Zheng S. L. , Genetic Disorders of Surfactant Protein Dysfunction: When to Consider and How to Investigate, Archives of Disease in Childhood. (Janauary 2017) 102, no. 1, 84–90, 10.1136/archdischild-2012-303143. 27417306

[15] Kurland G. , Deterding R. R. , Hagood J. S. et al., An Official American Thoracic Society Clinical Practice Guideline: Classification, Evaluation, and Management of Childhood Interstitial Lung Disease in Infancy, American Journal of Respiratory and Critical Care Medicine. (2013) 188, no. 3, 376–394, 10.1164/rccm.201305-0923ST. 23905526 PMC3778735

[16] Seidl E. , Carlens J. , Reu S. et al., Pulmonary Interstitial Glycogenosis—A Systematic Analysis of New Cases, Respiratory Medicine. (July 2018) 140, 11–20, 10.1016/j.rmed.2018.05.009. 29957271

[17] Magnani J. E. and Donn S. M. , Persistent Respiratory Distress in the Term Neonate: Genetic Surfactant Deficiency Diseases, Current Pediatric Reviews. (2020) 16, no. 1, 17–25, 10.2174/1573396315666190723112916. 31544695

[18] Nathan N. , Griese M. , Michel K. et al., Diagnostic Workup of Childhood Interstitial Lung Disease, European Respiratory Review. (February 2023) 32, no. 167, 10.1183/16000617.0188-2022. PMC9945877 36813289

[19] Bromley S. and Vizcaya D. , Pulmonary Hypertension in Childhood Interstitial Lung Disease: A Systematic Review of the Literature, Pediatric Pulmonology. (May 2017) 52, no. 5, 689–698, 10.1002/ppul.23632. 27774750

[20] McFetridge L. , McMorrow A. , Morrison P. J. , and Shields M. D. , Surfactant Metabolism Dysfunction and Childhood Interstitial Lung Disease (chILD), Ulster Medical Journal. (January 2009) 78, no. 1, 7–9. 19252722 PMC2629012

[21] Canakis A. M. , Cutz E. , Manson D. , and O’Brodovich H. , Pulmonary Interstitial Glycogenosis: A New Variant of Neonatal Interstitial Lung Disease, American Journal of Respiratory and Critical Care Medicine. (June 2002) 165, no. 11, 1557–1565, 10.1164/rccm.2105139. 12045133

[22] Deterding R. R. , Infants and Young Children With Children’s Interstitial Lung Disease, Pediatr Allergy Immunol Pulmonol.(March 2010) 23, no. 1, 25–31, 10.1089/ped.2010.0011. 22332029 PMC3269220

[23] Onland W. , Molenaar J. J. , Leguit R. J. et al., Pulmonary Interstitial Glycogenosis in Identical Twins, Pediatric Pulmonology. (October 2005) 40, no. 4, 362–366, 10.1002/ppul.20255. 16082691

[24] Castillo M. , Vade A. , Lim-Dunham J. E. , Masuda E. , and Massarani-Wafai R. , Pulmonary Interstitial Glycogenosis in the Setting of Lung Growth Abnormality: Radiographic and Pathologic Correlation, Pediatric Radiology. (September 2010) 40, no. 9, 1562–1565, 10.1007/s00247-010-1670-2. 20440487

[25] Deutsch G. H. and Young L. R. , Pulmonary Interstitial Glycogenosis: Words of Caution, Pediatric Radiology. (September 2010) 40, no. 9, 1471–1475, 10.1007/s00247-010-1730-7. 20593171

[26] King B. A. , Boyd J. T. , and Kingma P. S. , Pulmonary Maturational Arrest and Death in a Patient With Pulmonary Interstitial Glycogenosis, Pediatric Pulmonology. (November 2011) 46, no. 11, 1142–1145, 10.1002/ppul.21486. 21618718 PMC3832213

[27] Griese M. , Seidl E. , Hengst M. et al., International Management Platform for Children’s Interstitial Lung Disease (chILD-EU), Thorax. (March 2018) 73, no. 3, 231–239, 10.1136/thoraxjnl-2017-210519. 29056600

[28] Voss L. A. , Nevel R. J. , Wambach J. A. et al., ChILD Registry Collaborative. Genetic Testing Utilization in the U.S. Registry for Childhood Interstitial and Diffuse Lung Diseases, Pediatric Pulmonology. (April 2025) 60, no. 4, 10.1002/ppul.71073. PMC11960725 40167520

[29] Kornum J. B. , Christensen S. , Grijota M. et al., The Incidence of Interstitial Lung Disease 1995–2005: A Danish Nationwide Population-Based Study, BMC Pulmonary Medicine. (November 2008) 8, no. 1, 10.1186/1471-2466-8-24. PMC2642752 18983653

[30] Cunningham S. , Graham C. , MacLean M. et al., One-Year Outcomes in a Multicentre Cohort Study of Incident Rare Diffuse Parenchymal Lung Disease in Children (ChILD), Thorax. (February 2020) 75, no. 2, 172–175, 10.1136/thoraxjnl-2019-213217. 31748256

[31] Deutsch G. H. , Young L. R. , Deterding R. R. et al., Diffuse Lung Disease in Young Children: Application of a Novel Classification Scheme, American Journal of Respiratory and Critical Care Medicine. (December 2007) 176, no. 11, 1120–1128, 10.1164/rccm.200703-393OC. 17885266 PMC2176101

[32] Wert S. E. , Whitsett J. A. , and Nogee L. M. , Genetic Disorders of Surfactant Dysfunction, Pediatric and Developmental Pathology. (July-August 2009) 12, no. 4, 253–274, 10.2350/09-01-0586.1. 19220077 PMC2987676

[33] Sen P. , Yang Y. , Navarro C. et al., Novel FOXF1 Mutations in Sporadic and Familial Cases of Alveolar Capillary Dysplasia With Misaligned Pulmonary Veins Imply a Role for Its DNA Binding Domain, Human Mutation. (June 2013) 34, no. 6, 801–811, 10.1002/humu.22313. 23505205 PMC3663886

[34] Stankiewicz P. , Sen P. , Bhatt S. S. et al., Genomic and Genic Deletions of the FOX Gene Cluster on 16q24.1 and Inactivating Mutations of FOXF1 Cause Alveolar Capillary Dysplasia and Other Malformations, The American Journal of Human Genetics. (June 2009) 84, no. 6, 780–791, 10.1016/j.ajhg.2009.05.005. 19500772 PMC2694971

[35] Szafranski P. , Dharmadhikari A. V. , Wambach J. A. et al., Two Deletions Overlapping a Distant FOXF1 Enhancer Unravel the Role of lncRNA LINC01081 in Etiology of Alveolar Capillary Dysplasia With Misalignment of Pulmonary Veins, American Journal of Medical Genetics. (August 2014) 164, no. 8, 2013–2019, 10.1002/ajmg.a.36606. PMC4107046 24842713

[36] O′Reilly R. , Kilner D. , Ashworth M. , and Aurora P. , Diffuse Lung Disease in Infants Less Than 1 Year of Age: Histopathological Diagnoses and Clinical Outcome, Pediatric Pulmonology. (October 2015) 50, no. 10, 1000–1008, 10.1002/ppul.23124. 25603783

[37] Lipsett J. and Dishop M. K. , Strategies for the Neonatal Lung Biopsy: Histology to Genetics, Surgical Pathology Clinics. (December 2020) 13, no. 4, 657–682, 10.1016/j.path.2020.08.011. 33183726

[38] Greenhalgh R. M. , Yardley I. E. , Child F. , Bruce J. , and Humphrey G. M. , Lung Biopsy for Chronic Pulmonary Disease in Children, Journal of Pediatric Surgery. (July 2014) 49, no. 7, 1075–1077, 10.1016/j.jpedsurg.2013.10.026. 24952791

[39] Chan C. D. , Niyogi A. , Jaffray B. , Brodlie M. , and Gabra H. , Lung Biopsy in Children: When is it Useful?, Archives of Disease in Childhood. (March 2021) 106, no. 3, 291–293, 10.1136/archdischild-2019-318443. 32349979

[40] National Library of Medicine, National Center for Biotechnology Information, https://www.ncbi.nlm.nih.gov/clinvar/.

[41] Booysen L. , Interstitial Lung Disease in Infants Under 3 Months in a Developing Country, 2022, Stellenbosch University, https://scholar.sun.ac.za/server/api/core/bitstreams/9b13f694-7000-42d3-ae13-b218f4e97dc0/content, MMed (paed) research dissertation.

